# Nebulized heparin for inhalation injury in burn patients: a systematic review and meta-analysis

**DOI:** 10.1093/burnst/tkaa015

**Published:** 2020-06-04

**Authors:** Xiaodong Lan, Zhiyong Huang, Ziming Tan, Zhenjia Huang, Dehuai Wang, Yuesheng Huang

**Affiliations:** 1 Department of burn and plastic surgery, Chengdu Second People’s Hospital, Chengdu, 610021, China; 2 Department of Wound Repair, Institute of Wound Repair, Shenzhen People’s Hospital, the First Affiliated Hospital of South University of Science and Technology, and the Second Clinical Medical College of Jinan University, Shenzhen, 518020, China

**Keywords:** Burns, Inhalation injury, Heparin, Systematic review

## Abstract

**Background:**

Smoke inhalation injury increases overall burn mortality. Locally applied heparin attenuates lung injury in burn animal models of smoke inhalation. It is uncertain whether local treatment of heparin is benefit for burn patients with inhalation trauma. We systematically reviewed published clinical trial data to evaluate the effectiveness of nebulized heparin in treating burn patients with inhalation injury.

**Methods:**

A systematic search was undertaken in PubMed, the Cochrane Library, Embase, Web of Science, the Chinese Journals Full-text Database, the China Biomedical Literature Database and the Wanfang Database to obtain clinical controlled trails evaluating nebulized heparin in the treatment of burn patients with inhalation injury. Patient and clinical characteristics, interventions and physiological and clinical outcomes were recorded. Cochrane Risk of Bias Evaluation Tool and the Newcastle–Ottawa Scale were used to evaluate data quality. Potential publication bias was assessed by Egger’s test. A sensitivity analysis was conducted to assess the stability of the results. The meta-analysis was conducted in R 3.5.1 software.

**Results:**

Nine trials were eligible for the systematic review and meta-analysis. Nebulized heparin can reduce lung injury and improve lung function in burn patients with inhalation injury without abnormal coagulation or bleeding, but the findings are still controversial. Mortality in the heparin-treated group was lower than that of the traditional treatment group (relative risk (RR) 0.75). The duration of mechanical ventilation (DOMV) was shorter in the heparin-treated group compared to the traditional treatment group (standardized mean difference (SMD) −0.78). Length of hospital stay was significantly shorter than that in the traditional treatment group (SMD −0.42), but incidence rates of pneumonia and unplanned reintubation were not significantly different in the study groups (RRs 0.97 and 0.88, respectively). No statistically significant publication biases were detected for the above clinical endpoints (*p* > 0.05).

**Conclusions:**

Based on conventional aerosol therapy, heparin nebulization can further reduce lung injury, improve lung function, shorten DOMV and length of hospital stay, and reduce mortality, although it does not reduce the incidence of pneumonia and/or the unplanned reintubation rate.

Highlights

- Nebulized heparin can significantly reduce the comprehensive scores of lung injury, including on chest roentgenogram, oxygenation capacity, respiratory resistance, and compliance, among others, and does not cause coagulation disorders or changes in platelet count.
- Compared with patients in the traditional treatment group (N-acetylcysteine + Salbutamol), mortality was significantly reduced in the heparin-treated group (heparin + N-acetylcysteine + Salbutamol). And the duration of mechanical ventilation (DOMV) and length of hospital stay were significantly shortened.
- There were no significant differences in the incidence rates of pneumonia and unplanned reintubation between the heparin-treated and traditional treatment groups.

## Background

Inhalation injury-based respiratory failure is the main cause of death in patients with severe burns [1]. Smoke inhalation injury occurs through a variety of mechanisms, including direct thermal injury to the respiratory tract mucosa, and the type and extent of respiratory injury is influenced by the magnitude of exposure, the type and properties of toxic gases and chemicals constituting the smoke and the patient’s underlying respiratory function [2]. In general, damage from smoke inhalation results in airway edema and inflammation and, subsequently, cellular debris, mucus, fibrin clots and polymorphonuclear leukocytes combine to form casts that lead to ventilation/perfusion mismatch and poor oxygenation [3]. Moreover, coagulation is a part of the pathophysiological mechanism mediating smoke inhalation injury [4]. Activated inflammatory cells and cytokines potentially induce endothelial damage and increased vascular permeability, and the plasma exudate contains coagulation factors such as fibrinogen or prothrombin [5]. Furthermore, tissue factors expressed by pulmonary epithelial cells and alveolar macrophages initiate the extrinsic pathway of coagulation and may cause fibrin deposition in the alveolar space [6]. Fibrin formation and deposition in the alveolar space is considered a hallmark of smoke inhalation-induced acute lung injury and acute respiratory distress syndrome [7].

Heparin is a highly sulfated polyanionic glycosaminoglycan that has been traditionally used therapeutically for its anticoagulant activities [8]. However, existing evidence highlights the functional versatility of this molecule and its therapeutic potential outside of these traditional applications, especially in the control of inflammatory processes [9]. Heparin inhibits coagulation by providing fibrinolytic activation and inhibiting the early inflammatory response, thereby decreasing the histological score in acute lung injury [10]. Moreover, heparin regulates cell proliferation, prevents free radical-induced cell injury and is effective and safe for topical delivery in the lungs [11].

Nebulized therapies may combat the negative effects of inhalation injury by directly delivering medication to the lungs. Nebulized heparin has been used for the treatment of inhalation injury in burn patients for many years [2]. In animal experiments, nebulized heparin significantly alleviated inhalational lung injury, reduced the incidence of pneumonia and prevented coagulopathy [12–17]. However, clinical studies have shown that outcomes of nebulized heparin therapy are not consistent in burn patients with inhalation injury. We designed this meta-analysis to systematically evaluate the efficacy of nebulized heparin in the treatment of burn patients with inhalation injury and provide an evidence-based medical reference for the treatment of inhalation injury in burn patients.

## Methods

### Literature search

We undertook a search of PubMed, the Cochrane Library, Embase, Web of Science, the Chinese Journal Full-text Database, the China Biomedical Literature Database and the Wanfang Database with “inhalation injury”, “burn” and “heparin” as the search terms for articles published from database creation up to June 2019. The search strategy followed the protocol defined in the Cochrane System Evaluator’s Handbook (see online supplementary material for search strategies). Furthermore, the reference lists from included articles and relevant reviews were separately assessed to identify additional studies meeting the inclusion criteria of our study.

### Inclusion and exclusion criteria

The inclusion criteria were: (1) types of studies: clinical case–control trials, regardless of blinding; (2) subjects: burn patients with smoke inhalation injury, not limited by age and gender, wherein the heparin-treated group received nebulized heparin combined with bronchodilator or expectorant, and the conventional treatment group received bronchodilator or expectorant inhalation, with information on the start and duration of treatment, without limiting the form and dosage of each medicine; (3) effect endpoints: the physiological endpoints were arterial oxygen tension (PaO_2_), arterial oxygen tension to inspired oxygen concentration ratio (PaO_2_/FiO_2_), positive end-expiratory pressure (PEEP), chest roentgenogram, respiratory resistance and compliance, activated partial thromboplastin time (APTT), prothrombin time (PT) and platelet count, among others, whereas the clinical endpoints were mortality, duration of mechanical ventilation (DOMV), length of hospital stay, incidence of pneumonia and unplanned reintubation rate, among others; (4) publication language limited to Chinese and English; and (5) availability of sufficient data for a meta-analysis. The exclusion criteria were: (1) repeated publications; (2) reviews, preclinical studies, case reports, conference documents and irrelevant studies; (3) statistical data that could not be transformed and applied; and (4) inability to access the original full-text through various channels.

### Literature screening and data extraction

We used EndNote X9 to merge all documents and to eliminate duplicates. The remaining literature was initially screened by perusing headlines and abstracts. If the study was still unclear, the full text was read and further screened. Two researchers conducted the screening and data extraction based on the literature and differences, if any, were resolved by discussion with the third researcher. We extracted data using a data extraction table that we developed for this study. Data included research information (first author, year of publication, study design), object characteristics (sample size, age, region, and interventional measures), and results (PaO_2_, PaO_2_/FiO_2_, PEEP, chest roentgenogram, respiratory resistance and compliance, APTT, PT, platelet count, mortality, DOMV, length of hospital stay, incidence of pneumonia and unplanned reintubation rate).

### Quality evaluation

Two reviewers assessed the risk of bias individually for each study. The methodological quality assessment of randomized studies was undertaken with the Cochrane Risk of Bias Evaluation Tool [18]. The risk of bias was classified as low, unclear or high. The methodological quality of non-randomized studies was assessed by the Newcastle–Ottawa Scale (NOS) [19], which uses a grading system that ranges from 0 to 9 stars; studies of high quality were defined as those with scores greater than 6 stars.

### Statistical analysis

The measurement data are expressed as mean ± standard deviation (SD). The method described by Hou *et al*. was used to estimate the mean and SD using the median and extrema [20]. Meta-analysis was conducted using R 3.5.1 software. Relative risk (RR) and standardized mean difference (SMD) were used as the effect endpoints for the counting and measurement data and 95% confidence intervals (CIs) were calculated. Heterogeneity was tested with the 12 statistic. If 12 ≤ 50%, the research results were considered homogeneous and a fixed model was used for the meta-analysis; if 12 > 50% then there was heterogeneity among the research results and a random model was used for meta-analysis. Publication bias was determined by Egger’s test. If *p* < 0.05, there was publication bias, whereas *p* ≥ 0.05 indicated no publication bias. Furthermore, we conducted a sensitivity analysis to assess the stability of the results. All procedures of the present meta-analysis fulfilled the guidelines of the Preferred Reporting Items for Systematic reviews and Meta-Analyses (PRISMA; see online supplementary material for PRISMA checklist).

## Results

### Literature search and screening results

**Figure 1.**
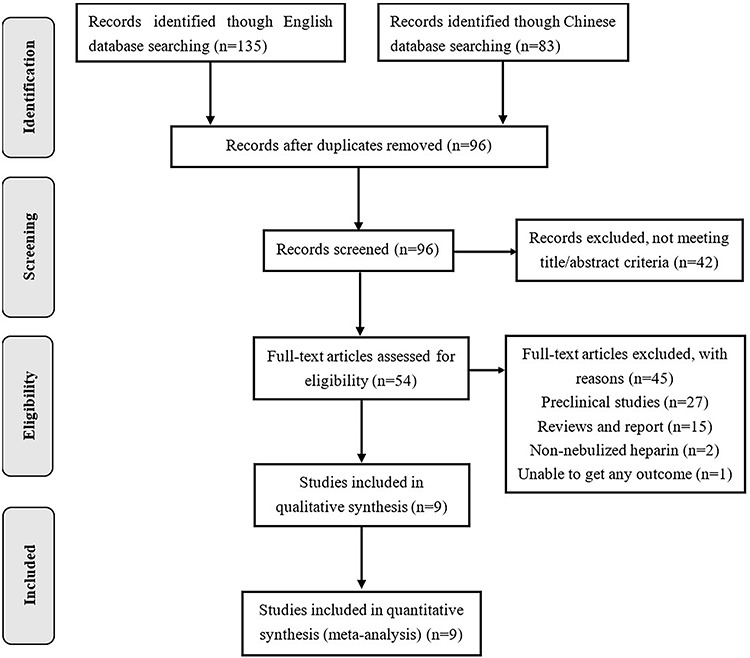
Preferred Reporting Items for Systematic reviews and Meta-Analyses (PRISMA) flow chart summarizing the results of the screening process and final article selections

The initial database searches yielded 218 relevant articles, and 83 articles were retrieved from the Chinese database. Manual searches of relevant references did not generate additional articles for inclusion in the meta-analysis. On checking for duplicates, 96 studies were excluded, and 84 articles were excluded after reviewing their title and abstract. Moreover, we excluded three articles that did not meet the inclusion criteria after reading 12 full texts. Ultimately, nine articles were eligible for inclusion in the meta-analysis, and none were multicenter clinical randomized controlled trials (Fig. 1).

### Characteristics and quality assessment of the eligible studies

The characteristics of the nine included studies are listed in Table 1. These nine studies were published between 1998 and 2019, and included a total of 609 burn patients with inhalation injury; the majority of included patients were from the USA. In the analysis dataset, 314 patients in the heparin-treated group received nebulized heparin combined with a bronchodilator or expectorant, whereas 295 patients received nebulized bronchodilator or expectorant in the traditional treatment group. The risk of bias assessment of one randomized study is illustrated in Table 2 and the methodological quality assessments of eight non-randomized trials, as determined by the NOS, are shown in Table 3.

**Table 1:** Summary of the studies included in systematic review and meta-analysis

Reference	Region	Patients (heparin/control)	Design	Agent (dosage)	Physiological endpoints	Clinical endpoints (heparin/control)
Holt (2008) [21]	Salt Lake City, Utah, USA	62/88 children or adults	Retrospective study using historical controls	Hep (5000 U，Q4h) + NAC + salbutamol，7 d	PaO_2_/FiO_2,_ optimal PaO_2_ =	Mortality (15/18), DOMV (18.2 ± 22.2/17.2 ± 18.1), LOHS (31.0 ± 22.2/31.9 ± 18.1), unplanned reintubation (9/7), pneumonia (39/44) =
Yip (2011) [22]	Singapore City, Singapore	52/11 adults	Retrospective study using historical controls	Hep (5000 U，Q4h) + NAC + salbutamol，7 d	APTT, PT, platelet count =	Mortality (19/6), DOMV (5.0 ± 20.0/9.0 ± 3.9), LOIC (6.0 ± 13.1/7.0 ± 3.5), pneumonia (9/2), bleeding (37/9) =
Sharma (2005) [23]	Indore, Madhya Pradesh, India	50/50 adults	Prospective study (single-center, double-blind)	Hep (5000 U，Q4h) + NAC + salbutamol，7 d	NR	Mortality (28/38), pneumonia (4/10) =
Desai (1998) [24]	Galveston, Texas, USA	43/47 children	Retrospective study using historical controls	Hep (5000 U，Q2h) + NAC，7 d	NR	Mortality (2/8), unplanned reintubation (3/12), LOHS (36.0 ± 21/48 ± 36.0), DOMV (3.4 ± 3.9/7.9 ± 3.3), pneumonia (20/30)↓
McIntire (2018) [25]	Birmingham, UK	36/36 adults	Retrospective study using historical controls	Hep (10,000 U，Q4h) + NAC + Salbutamol，28 d	NR	DOMV(7.0 ± 2.6/14.5 ± 4.3)↓,mortality (1/1), LOHS (17.0 ± 4.5/22.0 ± 6.2), pneumonia (23/26), bleeding (23/23) =
McGinn (2019) [26]	Auburn, Alabama, USA	22/26 adults	Retrospective study using historical controls	Hep (5000 U，Q4h) + NAC + salbutamol，5 d	NR	DOMV(3.0 ± 1.8/6.5 ± 3.6)↓, mortality (5/6), LOHS (12.4 ± 6.4/18.5 ± 9.0), unplanned reintubation (4/3), pneumonia (4/0) =
Miller (2009) [27]	Brooklyn, New York, USA	16/14 adults	Retrospective study using historical controls	Hep (10,000 U，Q4h) + NAC + salbutamol，7 d	Lung injury ↓	Mortality (1/6)↓
Kashefi (2014) [28]	Lubbock, Texas, USA	20/20 adults	Retrospective study using historical controls	Hep (5000 U，Q4h) + NAC + salbutamol，7 d	NR	Mortality (6/4), DOMV (8.5 ± 7.7/8.9 ± 11.2), LOHS (15.3 ± 10.8/16.3 ± 16.6) =, pneumonia (9/2) ↑
Rivero (2007) [29]	Tampa, Florida, USA	9/7 adults	Retrospective study using historical controls	Hep (10,000 U，Q4h) + NAC，7 d	Lung injury ↓	Mortality (1/3) ↓

↓ decrease, ↑ increase, = no difference

Counting data (n): mortality, unplanned reintubation, pneumonia, bleeding; measurement data (days): DOMV, LOHS, LOIC

Since physiological endpoints are dynamic indicators, we cannot provide specific numerical results. Lung injury included PaO2/FiO2, PaO2, PEEP, chest roentgenogram, respiratory resistance and compliance etc

*Hep* heparin, *NAC* N-acetylcysteine, *DOMV* duration of mechanical ventilation, *LOHS* length of hospital stay, *LOIC* length of intensive care, *APTT* activated partial thromboplastin time, *PT* prothrombin time, *PaO2* arterial oxygen tension, *PaO2/FiO2* arterial oxygen tension to inspired oxygen concentration ratio, *PEEP* positive end-expiratory pressure, *NR* no report

**Table 2:** Risk of bias assessment of randomized controlled trial

Study	Randomization	Allocation concealment	Blinding of participants	Incomplete outcome data	Selective outcome reporting	Other bias
Sharma (2005) [23]	Low	Unclear	Low	Low	Low	Unclear

**Table 3:** Quality assessment according to the Newcastle–Ottawa scale

Study	Selection	Comparability	Exposure	Total score
Holt (2008) [21]	3	1	3	7
Yip (2011) [22]	2	2	3	7
Desai (1998) [24]	2	2	3	7
McIntire (2018) [25]	3	1	2	6
McGinn (2019) [26]	3	2	3	8
Miller (2009) [27]	2	1	3	6
Kashefi (2014) [28]	3	2	3	8
Rivero (2007) [29]	3	1	3	7

### Results of meta-analysis

The predefined physiological and clinical endpoints were evaluated in the nine selected studies. Four of the studies reported the results of physiological endpoints. Because each study chose different endpoints, the methods varied widely. Furthermore, data synthesis was not available, and only qualitative descriptions were undertaken for these endpoints. Two clinical studies showed that nebulized heparin protected and improved lung function, which can significantly reduce the comprehensive score of lung injury, including oxygenation, chest roentgenogram, respiratory resistance and compliance [23,25]. One study reported the safety of nebulized heparin therapy, although the results showed this therapy did not cause systemic signs of coagulopathy, such as changes in APTT, PT and platelet count [22]. However, another study showed that burn patients with inhalation injury in the heparin-treated group did not experience effective and enduring improvements in oxygenation and other endpoints compared with the traditional treatment group [21]. The results of the clinical endpoints on the meta-analysis are as follows.

### Mortality

**Figure 2.**
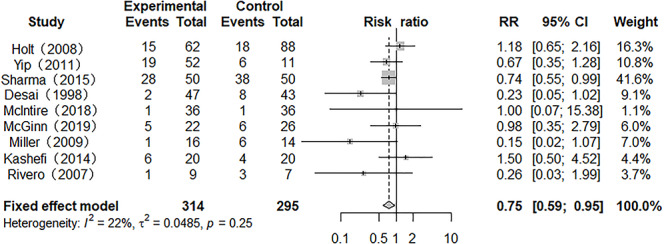
Forest plot of the effect of nebulized heparin on mortality in burn patients with inhalation injury. *RR* relative risk; *CI* confidence interval

All enrolled studies provided the main outcome: mortality. There was no heterogeneity between the studies (*p* = 0.25, *I*^2^ = 21.8%), and a fixed model was used for the meta-analysis. The pooled result indicated that the mortality of the heparin-treated group was lower than that of the traditional treatment group, with an RR of 0.75 (95% CI 0.59 to 0.95, *p* < 0.05) (Fig. 2).

### Duration of mechanical ventilation

**Figure 3.**
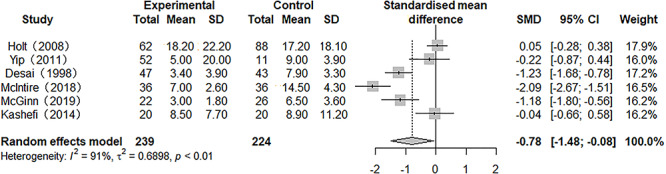
Forest plot of the effect of nebulized heparin on duration of mechanical ventilation in burn patients with inhalation injury. *RR* relative risk; *SMD* standardized mean difference; *CI* confidence interval

Six studies reported the results of DOMV, but there was heterogeneity between them (*p* = 0.09, *I*^2^ = 91.1%). A meta-analysis using a random model showed that the DOMV of patients treated with nebulized heparin was lower than that of patients treated with non-nebulized heparin, with an SMD of −0.78 (95% CI −1.48 to −0.08, *p* < 0.05) (Fig. 3).

### Length of hospital stay

**Figure 4.**
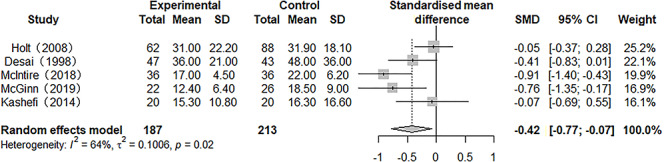
Forest plot of the effect of nebulized heparin on length of hospital stay in burn patients with inhalation injury. *RR* relative risk; *SMD* standardized mean difference; *CI* confidence interval

Five of the enrolled studies reported on the outcome of length of hospital stay. Heterogeneity was present (*p* = 0.02, *I*^2^ = 64.3%), and a random model was used for the meta-analysis. The results showed that the length of hospital stay of patients in the heparin-treated group was shorter than that in the traditional treatment group, and the SMD was −0.42 (95% CI −0.77 to −0.07, *p* < 0.05) (Fig. 4).

### Incidence of pneumonia

**Figure 5.**
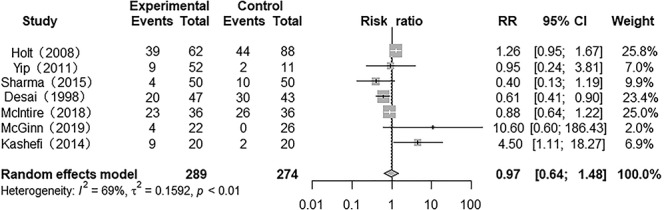
Forest plot of the effect of nebulized heparin on incidence of pneumonia in burn patients with inhalation injury. *RR* relative risk; *CI* confidence interval

Seven studies provided results for the incidence of pneumonia. There was heterogeneity among the studies (*p* = 0.00, *I*^2^ = 68.6%). The results of a meta-analysis in a randomized model indicated that the incidence of pneumonia was similar with interventions of nebulized heparin and conventional treatment (RR 0.97; 95% CI 0.64 to 1.48, *p* > 0.05) (Fig. 5).

### Unplanned reintubation

**Figure 6.**
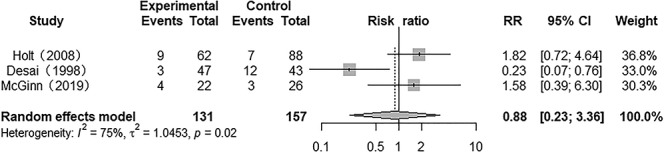
Forest plot of the effect of nebulized heparin on unplanned reintubation in burn patients with inhalation injury. *RR* relative risk; *CI* confidence interval

Three studies presented the results for unplanned reintubation and there was heterogeneity among them (*p* = 0.02, *I*^2^ = 75.0%). A random model was used for the meta-analysis, and results showed that the unplanned reintubation rate of patients in the heparin-treated group did not significantly differ from that of patients in the conventional treatment group (RR 0.88; 95% CI 0.23 to 3.36; *p* > 0.05) (Fig. 6).

### Sensitivity analysis and publication bias

A sensitivity analysis was used to assess the stability of the combined results. We found that the result for mortality may not be stable (see online supplementary material for forest plots of sensitivity analysis). This instability was caused by three studies [23, 24, 27]. Egger’s test was used to assess publication bias for mortality, DOMV, length of hospital stay, incidence of pneumonia and unplanned reintubation rate (*p* = 0.48, 0.36, 0.89, 0.67 and 0.77, respectively).

## Discussion

The main causes of combined burn and smoke inhalation injury are heat and smoke, wherein heat and chemicals associated with smoke can damage the respiratory tract and lung tissue, resulting in microthrombi formation, peroxidation and inflammatory reactions. These changes lead to narrowing or obstruction of the airway, pulmonary edema, atelectasis, pneumonia and other effects [3]. Therefore, based on the patient’s condition, the following actions are crucial to treating severe burns with inhalation injury: timely tracheotomy to provide mechanical ventilation, active control of excessive inflammation and other adverse reactions, reduction of secondary infection and promotion of airway and lung tissue repair [30]. In addition to anticoagulation, heparin has anti-inflammatory, anti-free radical, anti-infection, cytoproliferative and other effects [9]. Animal experiments have shown that, through its unique pharmacological effects, heparin can significantly reduce the degree of inhalation injury in lung tissues of experimental animals and can promote repair of damaged lung tissue, thereby ameliorating respiratory function and improving the survival rate [12–17]. Furthermore, nebulization significantly increases the bioavailability of heparin in airways and lung tissue and generally does not cause local bleeding and systemic coagulation disorders. Thus, nebulization is the preferred route of heparin administration in burn patients [11].

Some studies have explored the effect of nebulized heparin on inhalation injury in burn patients and in clinical controlled studies [21–29], although they were impaired by small sample sizes and the fact that most of the studies were retrospective clinical controlled trials. However, conflicting results have been presented from recent clinical trials. This study integrates nine clinically controlled studies evaluating the use of nebulized heparin in the treatment of burns patients with inhalation injury [21–29]. We systematically evaluated the difference in therapeutic effect between traditional nebulization therapy and heparin inhalation therapy for burn patients with inhalation injury and provided an evidence-based medical basis for inhalational heparin use in the treatment of burns with inhalation injury. The present meta-analysis of 609 burn patients with inhalation injury demonstrated the following.

First, nebulized heparin can significantly reduce the comprehensive scores of lung injury, including on chest roentgenogram, oxygenation capacity, respiratory resistance and compliance, among others [27, 29], and does not cause coagulation disorders or changes in platelet count [22]. Both clinical and methodological diversity precluded the combining of these studies in a meta-analysis. Only a narrative review of the literature is provided for physiological endpoints. Nonetheless, it is worth noting that PaO_2_/FiO_2_ and optimal PaO_2_ were unaffected by heparin treatment in the trials of Holt *et al*. [21], although the authors provided no reasonable explanation for such a result.

Second, compared with patients in the traditional treatment group, mortality was significantly reduced in the heparin-treated group and DOMV and length of hospital stay were significantly shortened. It is established that mortality, DOMV and length of hospital stay are important clinical outcomes in critically ill burn patients. The reduction in some or all outcomes between groups did not emerge in some of the included studies [21,22,25,26,28]. Perhaps this is attributable to the complex clinical condition of critically ill burn patients and the many facets, besides the management of inhalation injury, that can influence patient outcomes, including, but not limited to, wound care, operative planning and management of sepsis that likely significantly confound the interpretation of these outcomes. Therefore, the sample size may have been inadequate to detect a difference if one does exist. Furthermore, this could be one rationale to explain the different findings.

Third, there were no significant differences in the incidence rates of pneumonia and unplanned reintubation between the heparin-treated and traditional treatment groups. Pneumonia and unplanned reintubation are not directly related to the kind of medication used in patients with inhalation burn injury. Both presentations are associated with long-duration mechanical ventilation and extended intensive care unit (ICU) stay [31, 32]. Standardized clinical operations and care are decisive factors in eliminating these adverse consequences during mechanical ventilation [33]. We are unaware of any biologic basis to suggest that heparin is a pro-infectious or pro-inflammatory agent. However, Kashefi *et al*. found that heparin use resulted in a significant increase in pneumonia rates among burn patients [28]. They suspected, similarly to our view, that the reason for the increased infection rates was related to the frequent interruptions to the ventilator circuit and deficits in sterility during preparation and administration of the nebulized medication rather than from a direct effect of the medications themselves [28].

Four systematic researches of the medical literature have been recently published [34–37] that reviewed preclinical studies or clinical trials investigating the efficacy and safety of nebulized heparin in the setting of lung injury. Due to methodological diversity, they all mainly proceeded with a descriptive review, and the reviews of preclinical studies had similar results. Nebulized heparin can attenuate pulmonary coagulopathy and, frequently, inflammation in various models [35–37]. However, the conclusions of reviews of clinical trials were inconsistent. Similar to our findings, two previous systematic reviews reported nebulized heparin was beneficial and safe in acute lung injury. This intervention improved survival and decreased morbidity without altering systemic markers of anticoagulation [35,36]. However, a systematic review showed ambiguous results and emphasized concerns over the side effects of nebulized heparin, such as the spread of localized infections [37]. Some other results contradict our conclusions [34]. This may be attributed to major differences between the two studies, research subjects and data standardization. Our study focuses on the specific population of smoke inhalation injuries associated with burns. This facilitates the combining of clinical data to obtain objective meta-analytical results. More importantly, the results of the present systematic review expand our knowledge from these previous reviews because it identified and included several new articles.

Indeed, various factors, such as types of nebulizers used, heparin dosages, timing and frequency of nebulization, underlying pathologies of the studied patients, combined medicine, fluid resuscitation and so on, could have influenced the efficacy of nebulized heparin [10]. Elsharnouby *et al*. reported that, compared with patients who received heparin 5000 IU, nebulization with 10,000 IU heparin every 4 hours decreased lung injury scores and DOMV but had no effect on length of intensive care and mortality in adults with burn inhalation injury [38]. There was a sparsity of information with regard to the above items in the included studies, and we are unable to conduct a more specific meta-analysis on subgroups. Future studies should focus on some detailed issues, such as the optimal dosages and frequency of heparin nebulization.

Some limitations of this study should be noted. First, the small sample size might have affected the significant differences observed between the two study groups. Second, studies that included heparin and non-heparin interventions were a mix of RCTs and retrospective studies, primarily conducted in the USA. The combining of RCTs and retrospective studies in the present review were undertaken in compliance with methods outlined by the Cochrane Collaboration to include all relevant data from the literature [39]. However, regional differences may have contributed to the clinical heterogeneity. Third, in this meta-analysis, we were only able to analyse the data of 609 potentially eligible patients, as the authors of nine studies did not provide more underlying characteristic information of patients. Fourth, and not least, all selected studies were in English, and this could have conferred publication bias. At the same time, the sensitivity analysis results show that mortality may not be stable. We assumed that the large differences in sample size [27], research type [23] and research objects [24] may have had a greater impact on the analysis results. However, our results provide useful information, and larger-sample, multicenter, high-quality RCTs are needed to verify the outcomes of this meta-analysis.

## Conclusion

In summary, the current research evidence shows that nebulized heparin has a good effect in the treatment of burn patients with inhalation injury. Without affecting coagulation, this therapy can reduce lung damage, improve lung function, shorten the DOMV and length of hospital stay and reduce mortality, although it does not reduce the incidence of pneumonia and/or the unplanned reintubation rate. This study has certain positive reference value for the clinical treatment of burns with inhalation injury. Due to the related limitations, our findings need further confirmation by more clinical research with larger sample sizes.

## Abbreviations

PaO_2_: arterial oxygen tension; PaO_2_/FiO_2_: arterial oxygen tension to inspired oxygen concentration ratio; PEEP: positive end-expiratory pressure; APTT: activated partial thromboplastin time; PT: prothrombin time; DOMV: duration of mechanical ventilation; LOHS: length of hospital stay; NOS: Newcastle–Ottawa Scale; SD: standard deviation; RR: relative risk; SMD: standardized mean difference; CI: confidence interval; PRISMA: Preferred Reporting Items for Systematic reviews and Meta-Analyses

## Funding

This research did not receive any specific grant from funding agencies in the public, commercial or not-for-profit sectors.

## Availability of data and materials

The data used and/or analysed during the current study are accessible online.

## Authors’ contributions

Study design and drafting of this manuscript were undertaken by Xiaodong Lan, Ziming Tan and Yuesheng Huang. Literature retrieval and study selection were performed by Xiaodong Lan, Zhenjia Huang and Ziming Tan. Zhenjia Huang and Dehuai Wang performed quality evaluation of the study. Mathematical modeling and meta-analysis were conducted by Xiaodong Lan, Ziming Tan and Dehuai Wang. Results analysis and interpretation were undertaken by Xiaodong Lan, Zhiyong Huang and Dehuai Wang. The manuscript was drafted by Xiaodong Lan and Ziming Tan, and Yuesheng Huang contributed equally to this work. All authors have read and approved the final manuscript.

## Ethics approval and consent to participate

This study was exempt from institutional review board approval.

## Consent for publication

Not applicable.

## Conflicts of interest

None declared.

## Supplementary Material

Supplementary_File_1-Search_Strategy_tkaa015

Supplementary_File_2-PRISMA_Checklist_tkaa015

Supplementary_File_3-Sensitivity_Analysis_tkaa015
