# Supplementary material for: Nebulized heparin for inhalation injury in burn patients: a systematic review and meta-analysis
Source: Burns Trauma. 2020 Jun 4;8:tkaa015. doi: 10.1093/burnst/tkaa015 (PMC7271764; doi:10.1093/burnst/tkaa015)
Supplement: Supplementary_File_1-Search_Strategy_tkaa015 [file supplementary_file_1-search_strategy_tkaa015.docx]

Additional file 1

**Search Strategies by Database**

**Web of Science**

TOPIC: ("smoke inhalation injury" OR "inhalation injury") AND TOPIC: (burn OR burns) AND TOPIC: (heparin)

Timespan: All years. Indexes: SCI-EXPANDED, SSCI, A&HCI, CPCI-S, CPCI-SSH, BKCI-S, BKCI-SSH, ESCI, CCR-EXPANDED, IC.

Searched: 18/06/2019

Citations retrieved: 36

**PubMed**

("smoke inhalation injury" [mh] OR "inhalation injury" [tw]) AND (burns [mh] OR burn [tw]) AND (heparin [mh] OR heparin [tw])

Searched: 08/06/2019

Citations retrieved: 47

**Cochrane Library**

| ID | Search | Hits |
| --- | --- | --- |
| #1 | "smoke inhalation injury" OR "inhalation injury" | 125 |
| #2 | burn OR burns | 8436 |
| #3 | heparin | 11604 |
| #4 | #1 or #2 or #3 | 5 |

Searched: 10/06/2019 17:10:18

Citations retrieved:

0 Reviews: Cochrane Database of Systematic Reviews

0 Protocols: Cochrane Database of Systematic Reviews

5 Cochrane Central Register of Controlled Trials

**Embase**

| No. | Query | Results |
| --- | --- | --- |
| #1 | 'smoke inhalation injury'/exp OR 'smoke inhalation injury' OR 'inhalation injury'/exp OR 'inhalation injury' | 3945 |
| #2 | 'burn'/exp OR burn OR 'burns'/exp OR burns | 152740 |
| #3 | 'heparin'/exp OR heparin | 198991 |
| #4 | #1 AND #2 AND #3 | 140 |
| #5 | [humans]/lim AND [clinical study]/lim | 47 |

Searched: 01/06/2019

**Chinese Biomedical Literature Database**

Search: "Injury injury" [Common field: Smart] AND "Burn" [Common field: Smart] AND "Heparin" [Common field: Smart]

Duration: 1978-

Search result: 39

**Wanfang Database**

Search type: Inhalation injury * Burn * Heparin

Year range: unlimited

Search results: 7

**Chinese Journal Full-text Database**

Search: Full text = inhalation injury and full text = burn and full text = heparin (fuzzy match)

Year range: unlimited

Search results: 37
