# Supplementary material for: Nebulized heparin for inhalation injury in burn patients: a systematic review and meta-analysis
Source: Burns Trauma. 2020 Jun 4;8:tkaa015. doi: 10.1093/burnst/tkaa015 (PMC7271764; doi:10.1093/burnst/tkaa015)

Additional file 3

**Figure S1. Forest plots of Sensitivity analysis with comparisons of outcomes in meta-analysis.**

Mortality


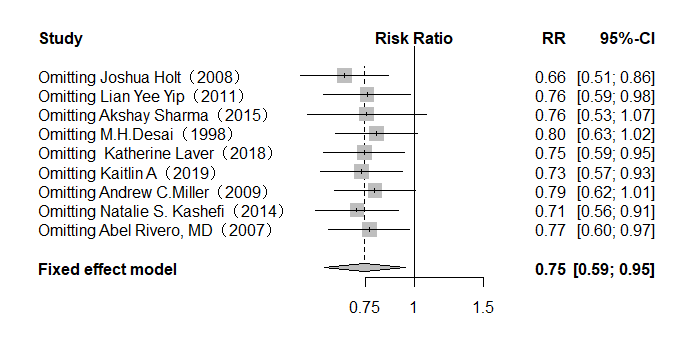


DOMV


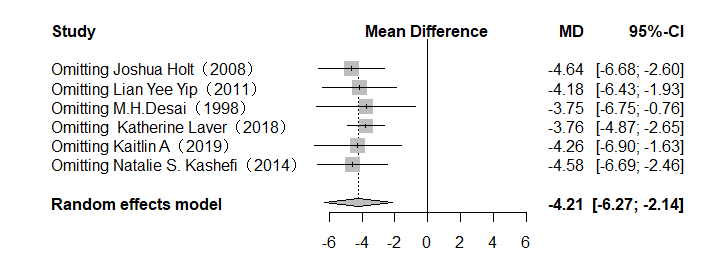


Length of hospital stay


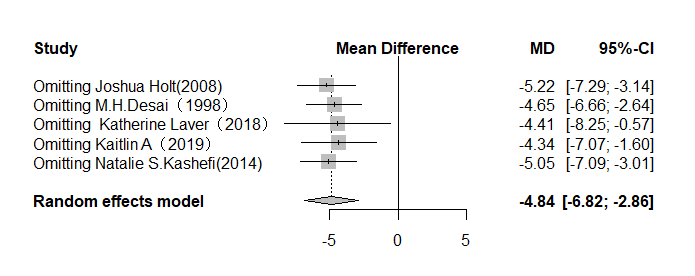


Incidence of pneumonia


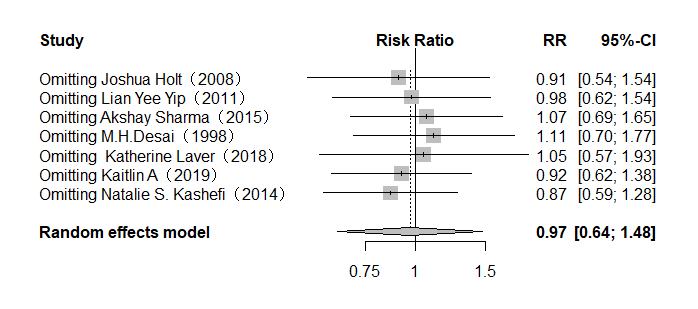


Unplanned reintubation


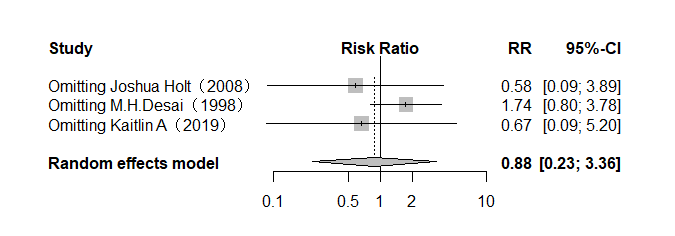

Supplement: Supplementary_File_3-Sensitivity_Analysis_tkaa015 [file supplementary_file_3-sensitivity_analysis_tkaa015.docx]
